# Identification of a Branch Number Locus in Soybean Using BSA-Seq and GWAS Approaches

**DOI:** 10.3390/ijms25020873

**Published:** 2024-01-10

**Authors:** Dongqing Dai, Lu Huang, Xiaoyan Zhang, Shiqi Zhang, Yuting Yuan, Gufeng Wu, Yichen Hou, Xingxing Yuan, Xin Chen, Chenchen Xue

**Affiliations:** Institute of Industrial Crops, Jiangsu Academy of Agricultural Sciences, Nanjing 210014, Chinazsqzsq126@163.com (S.Z.); y18075079765@163.com (Y.Y.); houyichen0324@163.com (Y.H.);

**Keywords:** soybean, branch number, GWAS, BSA, QTL

## Abstract

The determination of the soybean branch number plays a pivotal role in plant morphogenesis and yield components. This polygenic trait is subject to environmental influences, and despite its significance, the genetic mechanisms governing the soybean branching number remain incompletely understood. To unravel these mechanisms, we conducted a comprehensive investigation employing a genome-wide association study (GWAS) and bulked sample analysis (BSA). The GWAS revealed 18 SNPs associated with the soybean branch number, among which *qGBN3* on chromosome 2 emerged as a consistently detected locus across two years, utilizing different models. In parallel, a BSA was executed using an F_2_ population derived from contrasting cultivars, Wandou35 (low branching number) and Ruidou1 (high branching number). The BSA results pinpointed a significant quantitative trait locus (QTL), designated as *qBBN1*, located on chromosome 2 by four distinct methods. Importantly, both the GWAS and BSA methods concurred in co-locating *qGBN3* and *qBBN1*. In the co-located region, 15 candidate genes were identified. Through gene annotation and RT-qPCR analysis, we predicted that *Glyma.02G125200* and *Glyma.02G125600* are candidate genes regulating the soybean branch number. These findings significantly enhance our comprehension of the genetic intricacies regulating the branch number in soybeans, offering promising candidate genes and materials for subsequent investigations aimed at augmenting the soybean yield. This research represents a crucial step toward unlocking the full potential of soybean cultivation through targeted genetic interventions.

## 1. Introduction

Soybean is one of the most important economic crops and also serves as a primary source of plant-based protein and oilseeds, which are commonly employed in both human food and animal feed [1]. Because of constant increases in global population and the improvement of people’s living standards, the requirement for more soybeans has correspondingly increased. The genetic improvement of soybean varieties to enhance yields will become increasingly critical [2,3].

The strategies for increasing the production capabilities of soybeans include increasing the planting area of soybeans, encompassing approaches that intercrop with other crops, like maize, as well as planting soybeans on barren land [4]. Additionally, the related traits of yield components can be targeted. The former requires research on soybean stress, while the latter necessitates increased attention to the mechanism of soybean yield-related traits. Soybean presents as a typical pod crop, and its yield components are different from grain crops [5]. Soybean yield is governed by the pod number per plant, seed number per pod, and seed number. The number of branches directly affects the total pod number per plant in soybeans [6]. Branching plasticity reduces the branch number under dense planting, increasing the branch development relative to the land space per plant [7]. It is necessary to construct soybean varieties with an appropriate branch number according to typical cultivation practices.

The branch number is critical for increasing yield in soybeans, but its genetic regulatory mechanisms remain incompletely understood. Over the past decade, several approaches have been taken to characterize the genetic loci and genes responsible for branch numbers in soybeans. Using a recombinant inbred line (RIL) population for quantitative trait locus (QTL) is a typical method of characterizing the key loci related to the type in plants like legumes [8], rice [9], maize [10], and wheat [11]. Numerous branch number-related QTLs have been found in different linkage mapping populations [6,12,13,14,15,16,17,18]. With the development of the soybean reference genome and the increased usage of GWAS, some QTLs linked to soybean branching distributed on chromosomes 15, 17, 18, and 20 have been identified [19,20]. Shim et al. [6] performed QTL mapping for branch numbers with an RIL population and identified four related QTLs [6]. Two years later, they found a consistent QTL *GmBRC1* through GWAS and verified that *Glyma06g23410* is *GmBRC1*, which acts as a negative regulator of lateral branch development [21]. Sobhi et al. [22] determined that *Glyma.06G208900* acted as a candidate gene controlling the branch number [22]. Liang et al. [23] identified a predominant association locus on chromosome 18, *Dt2*, which confers soybean branch number in a natural population containing a total of 2409 soybean accessions, and demonstrated that *SoyZH13_18g242900* was a candidate gene for *Dt2* [23].

To further characterize the associated genes for the branch number in soybeans, we performed a comprehensive study integrating two methods: GWAS and BSA-seq. Our study involved an association panel of 301 soybean varieties for the GWAS and an F_2_ population of 30 individuals with the most branches and 30 individuals with the least branches for the BSA-seq. We ultimately mapped a branch number QTL across a specific chromosomal interval on chromosome 2 (12.16–12.42 Mb), encompassing 15 candidate genes. Among these genes, *Glyma.02G125200* and *Glyma.02G125600* emerged as robust candidates for controlling branch numbers in soybeans. The findings of this study will enable breeders to gain valuable insights for the purpose of selecting soybean germplasm resources that exhibit a corresponding branch number through the utilization of marker-assisted selection. Additionally, these findings will assist in identifying novel genes that play a role in regulating soybean branching.

## 2. Results

### 2.1. Evaluation of Branch Number in the Association Panel and F_2_ Population

**Figure 1:**
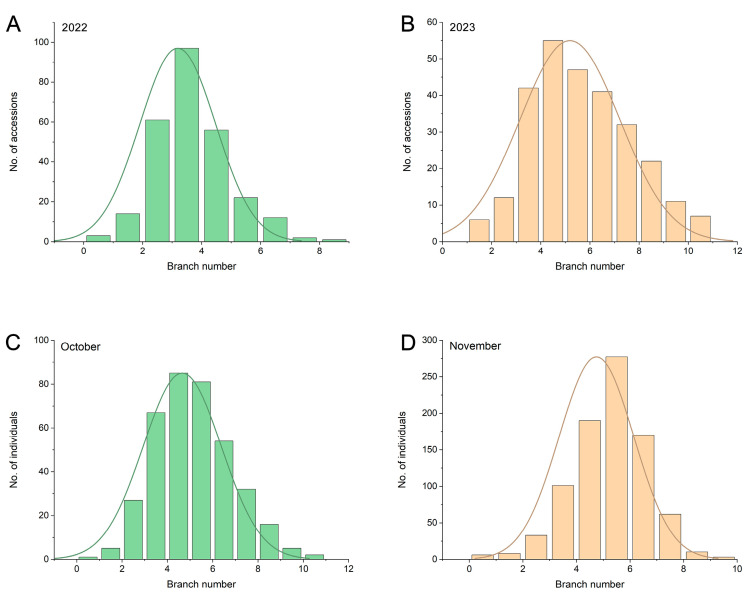
Frequency distribution of branch numbers. Frequency distribution of branch numbers in 2022 (**A**), and 2023 (**B**) throughout the association panel, and October (**C**), and November (**D**) throughout the F_2_ population. The curve indicates the standard normal curve based on the phenotypic distribution.

We analyzed the average branch number of each accession in the association mapping panel for 2022 and 2023. Continuous variation and an approximately normal distribution were identified (Figure 1A,B). The branch number ranged from 0.17 to 8.33 in 2022 and from 0.67 to 10.17 in the association panel, with mean values of 3.18 and 5.20, respectively. A higher coefficient of variation (CV) was identified in 2022, 40.25%, whereas a higher SD was found in 2023, 2.07 (Table 1 and Table S1). For the F_2_ segregating population, the branch numbers of the female parent Ruidou1 and male parent Wandou35 were 6.1 ± 0.66 and 0.7 ± 0.74, respectively (Figure S1). An ANOVA on the phenotypic data from natural soybean populations in 2022 and 2023 was conducted, and the findings indicated a significant difference between the two years (*p* < 0.01). The population was collected in October and November 2022 based on the maturity, with 860 individuals harvested in October and 375 individuals harvested in November. We examined the number of branches per plant individually, and observed a range from 0 to 10 in the F_2_ population, with an average number of 4.72. Continuous alterations were found in both batches (Figure 1A,B, Table S2). Extreme pools were chosen from among the 860 individuals.

### 2.2. Genomic Regions of Branch Number Identified by GWAS

**Figure 2:**
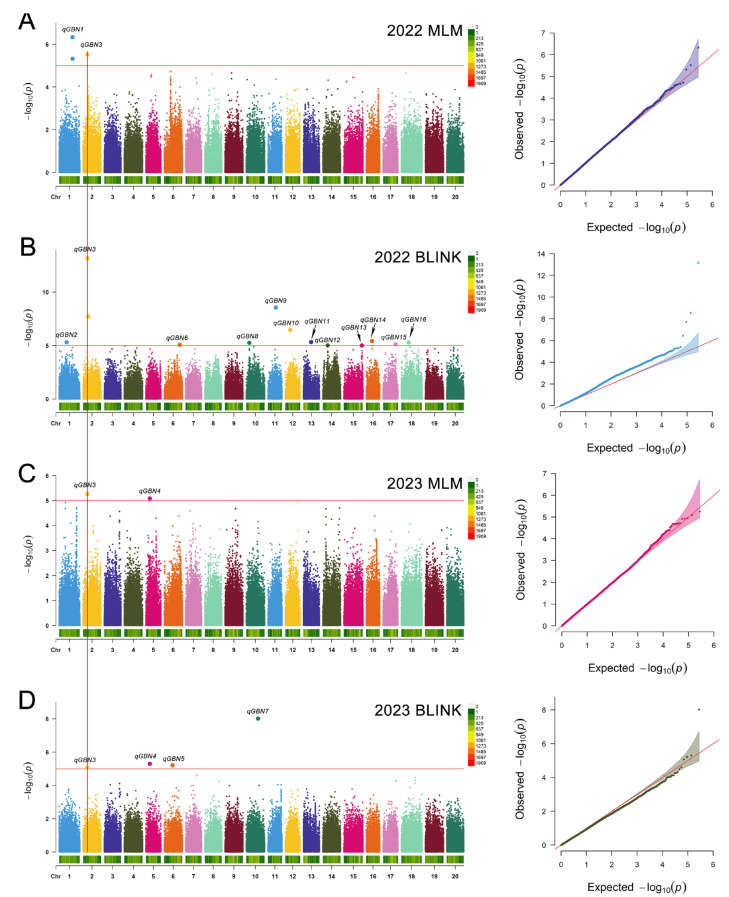
Manhattan and quantile–quantile plots of SNPs significantly linked to branch number using various models. (**A**) Bayesian information and Linkage-disequilibrium Iteratively Nested Keyway (BLINK) in 2022. (**B**) Mixed linear model (MLM) in 2022. (**C**) BLINK in 2023. (**D**) MLM in 2023.Manhattan plots: Different colors within the Manhattan plots represent different chromosomes across soybeans. The X-axis is the genomic position of the SNPs in the genome, and the Y-axis is the negative log base 10 of the *p*-values. The red horizontal line indicates the significance level. QQ plot: the Y-axis is the observed negative base 10 logarithm of the *p*-values, and the X-axis is the expected observed negative base 10 logarithm of the *p*-values. The red line represents the 45° centerline, and the gray area is the 95% confidence interval of the scattered points.

A total of 277,702 SNPs remained after filtering according to the selection criteria, which were employed to conduct GWAS for soybean branch numbers utilizing the MLM and BLINK models. According to a reasonable threshold (*p* ≤ 10^−5^), the GWAS revealed a total of 18 significant SNPs influencing the branch number, distributed on 13 chromosomes, including chromosomes 1, 2, 5, 6, 10, 11, 12, 13, 14, 15, 16, 17, and 18 (Table 2, Figure 2). Individual SNPs accounted for between 9.16 and 27.18% of the phenotypic variation. S02_1240704 and S02_1241353 were grouped based on the close proximity of the positions, much like S01_647060 and S01_647058. Therefore, a total of 16 QTLs were detected, noted as *qGBN1*-*qGBN16.* Among these QTLs, 13 were uniquely detected using only one approach, potentially due to environmental influences, three were co-identified when using different approaches, and *qGBN3* was detected both at two years, suggesting that *qGBN3* likely contained genes that regulate the number of soybean branches. The regions within 260 kb (12.16–12.42 M, Chr2) surrounding the S02_1240704 were employed as *qGBN3* based on the linkage disequilibrium (LD) decay determined previously. A total of 27 candidate genes, encompassing known or putative functions associated with signal transduction, amino acid metabolism, and translation, were identified in the region (Table S3).

### 2.3. BSA-Seq-Based Identification of Branch Number-Associated Genomic Regions

**Figure 3:**
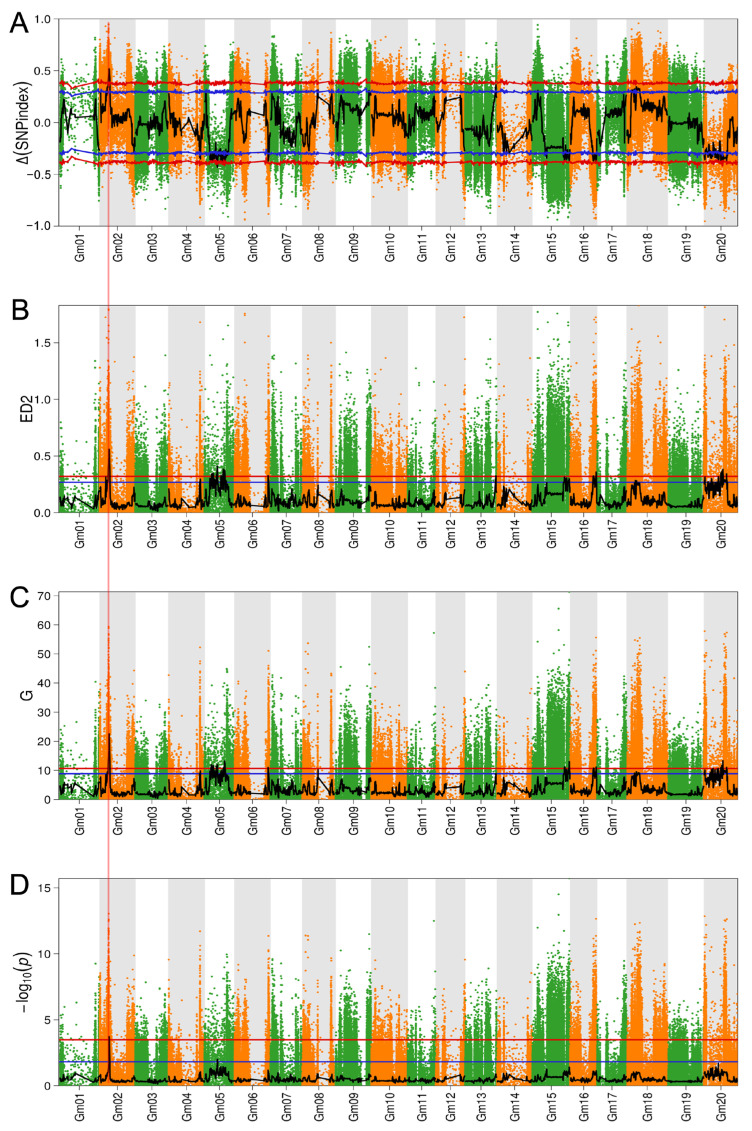
Information of candidate loci across ten chromosomes based on BSA results. Distribution of Δ(SNP-index) (**A**), ED2 value (**B**), G′ value (**C**) and log-transformed Fisher’s exact test *p*-value distribution, –log_10_(*p*) (**D**) on soybean chromosomes. The scatter plot represents the original values, green and orange are used to distinguish chromosomes, and the black curve represents the fitted values. The blue line represents the 95% confidence interval, and the red line represents the 99% confidence interval, respectively.

**Figure 4:**
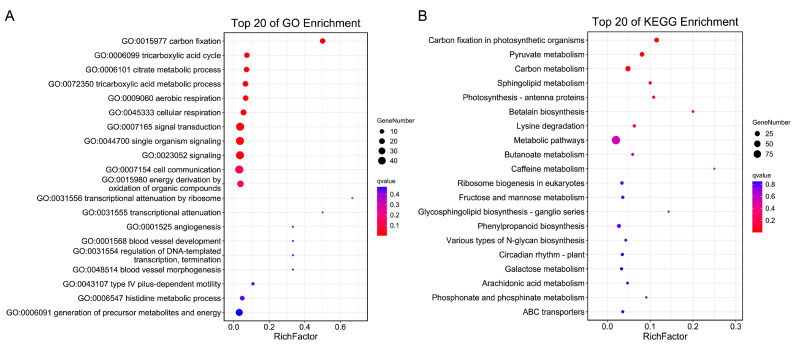
Gene enrichment analysis based on the BSA results. (**A**) Gene ontology enrichment using the top 20 significant terms. (**B**) KEGG pathway enrichment by using the top 20 significant pathways.

The bulked-segregant analysis coupled with the whole-genome sequencing (BSA-seq) was employed to characterize the branch number locus. Genomic DNA was extracted from 30 F_2_ plants with extremely high branch numbers (7–8), 30 F_2_ plants with extremely low branch numbers (0–3), and the two parents for the BSA-seq analysis. A total of 162 Gb of high-quality, clean data were acquired. The sequencing depths of the parents and extreme phenotypic pools were 30× and 20×, respectively (Table S4). The high-quality reads were aligned with the WM 82.a4 sequence. A total of 662,900 high-quality SNPs and 104,868 high-quality InDels following filtering were utilized for the subsequent analysis. Four strategies: Δ(SNP-index) (Figure 3A), ED (Figure 3B), G-value (Figure 3C), and Fisher’s exact test (Figure 3D) association analyses were employed, and 18 genomic regions across multiple chromosomes exceeded the threshold (99% confidence interval or *q*-value < 0.01). These QTL loci were termed *qBBN1*-*qBBN18*, and among them, six are distributed on chromosome 5, and five are distributed on chromosome 20. *qBBN1*, *qBBN3*, *qBBN4*, *qBBN6*, *qBBN7*, and *qBBN17* were identified through the use of three or more algorithms, and only *qBBN1* was detected through the use of all four methods (Table 3). An assessment of the WM 82.a4 sequence suggested that the 88 genomic regions contained 1109 genes. We conducted gene ontology (GO) enrichment and Kyoto Encyclopedia of Genes and Genomes (KEGG) pathway analysis of these corresponding genes. From the GO enrichment findings, we identified genes that were mainly involved in biological processes such as carbon fixation, cellular respiration, signal transduction, and cell communication (Figure 4A and Figure S2). According to our KEGG pathway analysis, the significantly enriched pathways throughout these genes included carbon fixation in photosynthetic organisms, pyruvate metabolism, and carbon metabolism (Figure 4B and Figure S3).

### 2.4. Candidate Loci Identified by GWAS and BSA

According to the GWAS and BSA analysis, we identified that the QTL loci *qGBN3* (12.16–12.42 Mb) characterized via GWAS and *qBBN1* (12.18–13.36 Mb) identified through BSA on chromosome 2 were co-located. This suggested that this interval has a robust linkage with the soybean branch number. Furthermore, we narrowed down the genomic interval to a 140 kb region (12.18–12.42 Mb). There are 15 putative genes annotated by the Soybase database (SoyBase, http://www.soybase.org/, accessed on 12 August 2023) (Table 4).

### 2.5. Candidate Gene Annotation and Expression Analysis

**Figure 5:**
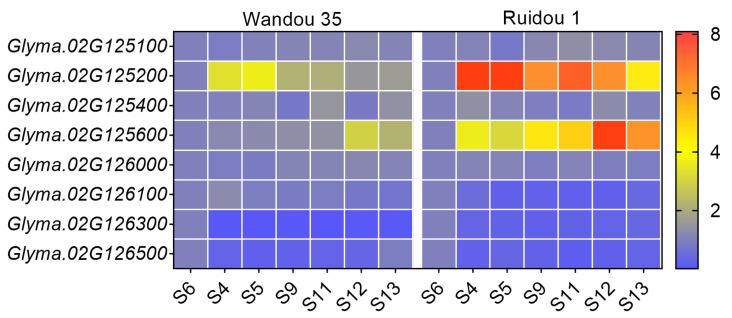
RT-qPCR analysis of candidate genes. RT-qPCR analysis of candidate genes throughout different tissues of Ruidou 1 and Wandou 35. S4–S13 indicates different developmental stages of soybeans, as outlined by Shen [24]. S4, leaf bud at the germination stage; S5, leaf bud at the trefoil stage; S6, leaf at the trefoil stage; S9, leaf bud at the flower bud differentiation stage; S11, flower bud at flower bud differentiation stage; S12, shoot meristem at flower bud differentiation stage; S13, flower bud at the flowering stage before flowering.

Based on the gene annotation, eight genes possessed functional annotations, while seven were unknown genes (Table 4). For instance, *Glyma.02G125100* encodes a putative 2-oxoglutarate/Fe(II)-dependent dioxygenase-like protein, *Glyma.02G125200* encodes a BHLH transcription factor, *Glyma.02G125600* was annotated to perform a role as an auxin synthesis-related gene, while *Glyma.02G126100* encodes a basic leucine zipper transcription (bZIP) factor-like protein. To characterize the significant gene influencing the branch number, we prioritized the genes with higher expression levels in the meristem-related tissues, as these genes may impact the formation and development of the soybean branches. The results of the RT-qPCR showed that *Glyma.02G125200* and *Glyma.02G125600* were highly expressed in the axillary bud tissues, especially in Wandou 35 (Figure 5). This indicates that *Glyma.02G125200* and *Glyma.02G125600* may have critical functions in branch development.

## 3. Discussion

The aerial segment of soybeans [*Glycine max* (L.) Merr.] is made up of the main stem and a variable number of branches, with leaves, flowers, and pods attached [13]. The number of soybean branches is important for the morphogenesis of soybean plants and is closely tied to the plant lodging resistance as well as the yield per plant. Moreover, the branch number can influence the population seed yield by impacting light utilization and ventilation. The extent of branching depends on various influences exerted by the growth environment, encompassing nutritional conditions, planting pattern, planting date, and, particularly, plant density [13,25]. The yield of various soybean varieties differs significantly under various planting densities [26]. Under low density, multi-branched varieties can acquire a high yield through increased branching, while under high density, branching decreases [22]. To remove the influence of density on soybean branching, all experiments in our study were carried out using a low and uniform density of 83,333 plants/ha. The branch number of the soybeans in the associated population was considerably variable (Figure 1).

Soybean branching is modulated by an intricate spatial–temporal mechanism, controlling the outgrowth of axillary buds following the initiation of axillary meristems [27]. An increasing number of QTLs regulating branch development have been characterized through the use of various mapping populations in soybeans [6,12,13,18]. However, the identified QTLs span various plausible genes due to the limited number of molecular markers and uneven distributions. The reference genome of a cultivated accession (Williams 82) was released in 2010 [28]. With the emergence and development of high-throughput sequencing technology alongside its high efficiency, the GWAS and BSA-seq methods have been widely employed in the analysis of the critical agronomic characteristics of various crops.

By integrating GWAS and BSA, we identified a novel QTL on chromosome 2 associated with the soybean number, *qGBN3*/*qBBN1*, which has been detected by different MLM and BLINK models over two years and four BSA algorithms (Figure 2 and Figure 3). This suggested that this overlap might be a necessary genetic component accounting for the number of soybean branches. We further narrowed the region of the co-located QTL to a precise range of 140 kb (12.18–12.42 Mb, Chr2). Moreover, the genome regions overlapping between this study and previous research were determined. These regions included *qBBN8* (47.02–48.27 Mb, Chr6) and *qGBN6* (44.28–44.48 Mb, Chr6), which were included within the *Qsb 6* (40.46–48.30 Mb, Chr6); *qGBN9* (8.49–22.59 Mb, Chr11) was similar to *qBR11-1* (10.83–25.08 Mb, Chr11) [6,12]. This corroborated the accuracy and reliability of the correlation examination in this study.

Integrating the gene annotation and expression experiments, two candidate genes were considered to be potential candidates for regulating the soybean branch numbers. *Glyma.02G125200* encodes a Basic Helix-Loop-Helix (bHLH) transcription factor known as bHLH49. The bHLH transcription factor family is one of the largest families of transcription factors that plays a crucial role in plant development. The loss of function of the bHLH homolog LAX1 in rice results in the abortion of panicle branching [29], indicating that *Glyma.02G125200* may play a role in soybean branching. *Glyma.02G125600* encodes an Indole-3-acetic acid-amido synthetase, known as GH3.1, which was involved in the plant hormone signal transduction pathway via KEGG annotation analysis. Gretchen Hagen3 (GH3) has dual roles in plant development and responses to biotic or abiotic stress [30]. The overexpression of the homologous genes, *CsGH3.1* and *CsGH3.1L* in citrus resulted in the significant downregulation of the expression levels of the annotated auxin/indole-3-acetic acid family genes and elevated branching [31]. This suggests that auxin synthesis and transduction may be crucial for promoting the branch number of soybeans.

Notably, the expression level of *Glyma.02G126100*, encoding a bZIP family transcription factor in the meristematic tissues of Ruidou 1, was lower than in Wandou 35 (Figure 5). The bZIP domain transcription factors play an important role in plant external stress and development [32,33]. We could not exclude that *Glyma.02G126100* operated as a candidate gene, which may have a negative regulatory influence on soybean branching. Further experiments, including gene editing and overexpression, will be necessary to confirm the function of these genes in the future.

This study represents a comprehensive contribution to our understanding of the genetic mechanism underlying branch number and its influence on the soybean yield. Identifying novel loci and candidate genes using a combination of GWAS and BSA allows for the development of a theoretical foundation for future studies on the genetic regulation of soybean branching. Overall, this study will be valuable in breeding high-yield soybeans and global food and oil security.

## 4. Materials and Methods

### 4.1. Plant Materials and Phenotypic Evaluation

A total of 301 soybean accessions from different Chinese provinces were collected to construct an association mapping panel. All materials were grown in Nanjing, Jiangsu Province, in 2022 and 2023. The experiment utilized a randomized complete block design with a single-row plot and three replicates. Each plot consisted of a single row, which was 3 m long, with 40 cm between rows and approximately 30 cm between plants. An F_2_ population composed of 1235 lines was developed from cross-breeding between Ruidou 1, a high branching number cultivar, and Wandou 35, with a low branch number. This F_2_ population and their parents were grown in Nanjing, Jiangsu province, in the summer of 2022. Again, the row length, spacing, and plant interval were 3 m, 40 cm, and 30 cm, respectively. The edge plants were removed, and the branch number of each plant was recorded as the number of effective branches upon the main stem with two or more nodes and at least one mature seed pod at harvest. For the association mapping panel, the average branch number of the six plants was obtained as the branch number of each accession. For the F_2_ population, we investigated 1235 individual soybean plants.

### 4.2. Statistical Analysis

In this study, SAS 9.2 software (SAS Software, Cary, NC, USA) was used to perform the Student’s *t*-test, and ANOVA was used to determine the significance differences of the phenotypic data from natural soybean populations in 2022 and 2023.

### 4.3. DNA Extraction and Whole-Genome Resequencing

For the F_2_ population, 30 individuals with the most branch numbers and 30 with the least branch numbers were chosen to develop two DNA mixed pools named “M_pool” and “F_pool,”, respectively. The “M pool” and “F pool” were developed by mixing equal amounts of high-quality DNA extracted from each individual leaf. The sequencing depth of mixed pools and parents were 50× and 30×, respectively.

The DNA libraries were sequenced using the Illumina sequencing platform by Genedenovo Biotechnology Co., Ltd. (Guangzhou, China). High-quality clean reads were contrasted with the reference genome utilizing BWA 0.7.1 [34]. The reference genome employed was the Glycine_max_v4.0 version of Williams 82 (https://www.ncbi.nlm.nih.gov/assembly/GCF_000004515.6/, accessed on 10 February 2023). To detect SNP and insertion/deletion (InDel) variants, we utilized GATK software v4.1 [35], and ANNOVAR software [36] to perform variant annotation and predict variant impact.

### 4.4. Genome-Wide Association Analyses

The genotypic data from 301 soybean materials were outlined in our previous study (not yet published). A total of 277,022 filtered high-quality SNPs spanning the entire genome, with a minimum allele frequency (MAF) ≥ 0.05, were employed to conduct the GWAS. The GWAS was performed using the mixed linear model (MLM) and Bayesian-information and Linkage-disequilibrium Iteratively Nested Keyway (BLINK) method, [37], using TASSEL 3.0 and GAPIT 3.0 [38,39]. At *r*^2^ = 0.1, the mean LD decay was 100 kb throughout all the chromosomes. A significance threshold of 1 × 10^−5^ was established to characterize significant associations [40]. Manhattan and quantile–quantile (QQ) plots were generated using the “CMplot” package in the R environment [41].

### 4.5. Bulk Segregant Sequencing Analyses

Four widely used analysis methods in BSA analysis were utilized, including Δ (SNP-index) statistics [42], the Euclidean distance (ED) algorithm [43], the G statistic, and the Fisher exact test [44,45]. The four methods we utilized were all based on a 1000 kb sliding window with a step size of 10 kb, applied to calculate the average and smooth the map. A 99% confidence level was chosen as the threshold for screening, and the window above the confidence level was defined as the area linked to the branch number. The intervals obtained using the four correlation analysis approaches were compared, and the overlapping interval was the QTL interval associated with the branch number. The genes and polymorphic sites in the candidate interval were annotated utilizing the website https://www.soybase.org/ (accessed on 12 August 2023).

### 4.6. Candidate Genes Identification and Description

In our study, the same intervals of BSA-seq and GWAS were the co-located QTL, and Soybase (https://www.soybase.org/, accessed on 29 November 2023) was then employed for gene annotation. Candidate genes were identified based on their expression patterns and homolog data of all genes within the co-located QTL.

### 4.7. Analyses of Gene Expression Patterns

Total RNA was extracted from the leaves and meristems of Ruidou 1 and Wandou 35 using TRIzol [46]. In total, seven RNA samples, including leaf at the trefoil stage (S6), leaf bud at the germination stage (S4), at the trefoil stage (S5), and at the flower bud differentiation stage (S9), flower bud (S11) and shoot meristem (S12) at flower bud differentiation stage, and flower bud at the flowering stage before flowering (S13) were acquired [24]. Three biological replicates were conducted, with the grains derived from three plants werew combined into one biological replicate. The primers used for RT-qPCR are listed in Table S5.

## 5. Conclusions

In this study, we utilized two methods, GWAS analysis and BSA seq, to co-locate a branch number QTL qGBN3/qBBN1 situated on chromosome 2. By conducting a functional annotation analysis and gene expression analysis on 15 candidate genes across the candidate region, we found two candidate genes, *Glyma.02G125200* and *Glyma.02G125600*. Further functional analysis of these two genes will be conducted to uncover the regulatory mechanism of this gene in the soybean branch number.

## Figures and Tables

**Table 1:** Descriptive statistics for branch number of soybeans across the association mapping panel.

Year	Mean	SD	CV (%)	Min	Max	Kurt	Skew
2022	3.18	1.28	40.25	0.17	8.33	0.93	0.64
2023	5.20	2.07	39.82	0.67	10.17	0.64	0.93

**Table 2:** QTL and SNPs significantly linked to soybean branch number.

QTL	SNP	Chromosome	Position	*p* Value	R^2^	Allelic Variation	Year	Method
*qGBN1*	S01_647060	1	37500028	4.69 × 10^−7^	11.65	C/T	2022	MLM
	S01_647058	1	37499994	4.79 × 10^−6^	9.82	T/C	2022	MLM
*qGBN2*	S11_383602	1	20996571	5.00 × 10^−6^	9.51	T/A	2022	BLINK
*qGBN3*	S02_1240704	2	12264757	6.94 × 10^−14^	27.18	A/G	2022	BLINK
	S02_1241353	2	12324064	1.87 × 10^−8^	14.17	C/T	2022	BLINK
	S02_1240704	2	12264757	2.99 × 10^−6^	9.77		2022	MLM
	S02_1240704	2	12264757	5.56 × 10^−6^	9.32		2023	MLM
	S02_1240704	2	12264757	8.57 × 10^−6^	9.25		2023	BLINK
*qGBN4*	S05_3893779	5	11122603	5.02 × 10^−6^	9.55	T/C	2023	BLINK
	S05_3893779	5	11122603	8.31 × 10^−6^	9.27		2023	MLM
*qGBN5*	S06_4772142	6	23576358	6.07 × 10^−6^	9.66	C/A	2023	BLINK
*qGBN6*	S06_5137235	6	44380411	8.54 × 10^−6^	9.25	G/A	2022	BLINK
*qGBN7*	S10_8203320	10	33337627	9.46 × 10^−9^	14.86	G/T	2023	BLINK
*qGBN8*	S10_7804544	10	8551686	5.73 × 10^−6^	9.30	A/T	2022	BLINK
*qGBN9*	S11_8849037	11	22593147	2.81 × 10^−9^	15.85	T/A	2022	BLINK
*qGBN10*	S12_9388376	12	13737593	3.51 × 10^−7^	12.17	C/T	2022	BLINK
*qGBN11*	S13_10274380	13	21682594	4.83 × 10^−6^	9.82	G/A	2022	BLINK
*qGBN12*	S14_10921879	14	13946264	9.64 × 10^−6^	9.25	G/A	2022	BLINK
*qGBN13*	S15_12533199	15	50770118	9.67 × 10^−6^	9.16	A/G	2022	BLINK
*qGBN14*	S16_12903158	16	16529439	3.82 × 10^−6^	10.02	G/C	2022	BLINK
*qGBN15*	S17_13967003	17	35344266	7.99 × 10^−6^	9.27	C/A	2022	BLINK
*qGBN16*	S18_14535964	18	20984058	5.30 × 10^−6^	9.51	A/G	2022	BLINK

**Table 3:** QTLs linked to branch numbers identified using BSA-seq based on four methods.

QTLs	Chromosome	Start Position (bp)	End Position (bp)	Peak	Method
*qBBN1*	Gm02	11420001	15020000	0.559684	ED
	Gm02	11440001	15070000	22.53829	Gst
	Gm02	11530001	15020000	0.517047	Δ(SNP-index)
	Gm02	12280001	13360000	0.003283	Fisher
*qBBN2*	Gm05	8580001	10660000	11.91817	Gst
	Gm05	9270001	10430000	0.340816	ED
*qBBN3*	Gm05	11450001	12970000	11.65613	Gst
	Gm05	11460001	12850000	0.348614	ED
	Gm05	11570001	12570000	−0.37708	Δ(SNP-index)
*qBBN4*	Gm05	16210001	18710000	11.9553	Gst
	Gm05	16210001	18940000	0.411016	ED
	Gm05	16210001	18910000	−0.4334	Δ(SNP-index)
*qBBN5*	Gm05	22600001	23850000	−0.37968	Δ(SNP-index)
	Gm05	22830001	23880000	0.329834	ED
*qBBN6*	Gm05	25240001	27230000	0.383032	ED
	Gm05	25740001	26750000	−0.39135	Δ(SNP-index)
	Gm05	26070001	27230000	10.96294	Gst
*qBBN7*	Gm05	27540001	29280000	0.374127	ED
	Gm05	27850001	29330000	13.24474	Gst
	Gm05	27850001	29220000	−0.41289	Δ(SNP-index)
*qBBN8*	Gm06	47020001	48270000	11.02317	Gst
	Gm06	47060001	48240000	0.326973	ED
*qBBN9*	Gm13	43170001	44200000	0.325159	ED
*qBBN10*	Gm15	44910001	46480000	11.18481	Gst
*qBBN11*	Gm15	51360001	53140000	13.02159	Gst
*qBBN12*	Gm16	32020001	33890000	0.324992	ED
	Gm16	32490001	34130000	10.81712	Gst
*qBBN13*	Gm16	36050001	37470000	0.35877	ED
	Gm16	36170001	37440000	11.15172	Gst
*qBBN14*	Gm20	19840001	20840000	−0.38348	Δ(SNP-index)
*qBBN15*	Gm20	23160001	25850000	0.361823	ED
	Gm20	23330001	24470000	10.97986	Gst
*qBBN16*	Gm20	24620001	25850000	11.71612	Gst
	Gm20	24620001	25660000	−0.39685	Δ(SNP-index)
*qBBN17*	Gm20	25860001	27730000	0.381719	ED
	Gm20	26020001	27610000	13.35137	Gst
	Gm20	26170001	27320000	−0.39762	Δ(SNP-index)
*qBBN18*	Gm20	31420001	32420000	11.04967	Gst
	Gm20	31420001	32420000	0.341994	ED

**Table 4:** Annotated genes from GWAS and BAS-seq co-located intervals.

GeneID	Start Position (bp)	End Position (bp)	Symbol	Annotation
*Glyma.02G125100*	12220107	12224403	*SRG1*	2-oxoglutarate/Fe(II)-dependent dioxygenase-like
*Glyma.02G125200*	12226179	12230868	*BHLH49*	Transcription factor bHLH49 isoform X1
*Glyma.02G125300*	12229902	12230859	-	-
*Glyma.02G125400*	12246600	12253822	*WIT2*	WPP domain-interacting tail-anchored protein 2-like isoform X4
*Glyma.02G125500*	12268030	12268302	-	-
*Glyma.02G125551*	12279258	12279410	-	-
*Glyma.02G125600*	12302237	12304986	*GH3.1*	Indole-3-acetic acid-amido synthetase GH3.1
*Glyma.02G125700*	12306697	12309236	-	-
*Glyma.02G125800*	12314438	12315067	-	-
*Glyma.02G125900*	12317269	12318465	-	-
*Glyma.02G126000*	12321542	12323547	*IRT2*	Fe(2+) transport protein 1
*Glyma.02G126100*	12368440	12371047	*BZIP43*	Basic leucine zipper transcription factor-like protein
*Glyma.02G126200*	12375750	12376682	-	-
*Glyma.02G126300*	12388195	12394795	*CPN60B4*	RuBisco large subunit-binding protein subunit beta
*Glyma.02G126500*	12412320	12413154	*SNAT2*	Serotonin N-acetyltransferase 2, chloroplastic

## Data Availability

The data presented in this study are available on request from the corresponding author.

## Supplementary Materials

The following supporting information can be downloaded at https://www.mdpi.com/article/10.3390/ijms25020873/s1.
