# Supplementary material for: Identification of a Branch Number Locus in Soybean Using BSA-Seq and GWAS Approaches
Source: Int J Mol Sci. 2024 Jan 10;25(2):873. doi: 10.3390/ijms25020873 (PMC10815202; doi:10.3390/ijms25020873)
Supplement: Supplementary file 1 [file ijms-25-00873-s001.zip › ijms-2775726-supplementary.pdf]

Table S1 Descriptive statistics for branch number in F_2 population.

Harvest	Mean	SD	CV(%)	Min	Max	Kurt	Skew
October	4.747674	1.39442	29.37059	0	9	0.593704	-0.32056
November	4.656	1.724799	37.04465	0	10	-0.02108	0.3565
All	4.719838	1.502312	31.82973	0	10	0.379312	-0.03304

Table S2 Genes associated with branch number identified using GWAS.

GeneID	Symbol	Start(bp)	End(bp)	Description
Glyma.02G124000	*At2g03980*	12102056	12105040	GDSL esterase/lipase
Glyma.02G124100	*STP1*	12108949	12112329	sugar carrier protein C
Glyma.02G124200	--	12124496	12126206	hypothetical protein glysoja_014752
Glyma.02G124300	*MYB92*	12143469	12145413	transcription factor MYB92
Glyma.02G124400	*RAE1*	12154608	12161088	unknown
Glyma.02G124500	*HSP90-5*	12162865	12171161	Heat shock protein 90-5, chloroplastic isoform B
Glyma.02G124600	*prxl2a*	12178000	12181865	uncharacterized protein
Glyma.02G124700	*SRG1*	12184046	12187279	SRG1
Glyma.02G124800	*PPME1*	12189727	12191734	putative pectinesterase 63
Glyma.02G124900	*SRG1*	12196992	12199411	SRG1
Glyma.02G125000	*SRG1*	12202873	12208105	Protein SRG1-like protein
Glyma.02G125100	*SRG1*	12220107	12224403	SRG1
Glyma.02G125200	*BHLH49*	12226179	12230868	transcription factor bHLH49 isoform X1
Glyma.02G125300	--	12229902	12230859	hypothetical protein
Glyma.02G125400	*WIT2*	12246600	12253822	hypothetical protein
Glyma.02G125500	--	12268030	12268302	hypothetical protein D0Y65_003732, partial
Glyma.02G125551	--	12279258	12279410	-
Glyma.02G125600	*GH3.1*	12302237	12304986	probable indole-3-acetic acid-amido synthetase GH3.1
Glyma.02G125700	--	12306697	12309236	hypothetical protein glysoja_014737
Glyma.02G125800	--	12314438	12315067	hypothetical protein GLYMA_02G125800
Glyma.02G125900	--	12317269	12318465	uncharacterized protein
Glyma.02G126000	*IRT2*	12321542	12323547	fe(2+) transport protein 1
Glyma.02G126100	*BZIP43*	12368440	12371047	basic leucine zipper 43
Glyma.02G126200	--	12375750	12376682	phytosulfokines 1
Glyma.02G126300	*CPN60B4*	12388195	12394795	ruBisCO large subunit-binding protein subunit beta
Glyma.02G126500	*SNAT2*	12412320	12413154	serotonin N-acetyltransferase 2, chloroplastic
Glyma.02G126600	--	12432790	12434857	uncharacterized protein

Table S3 DNA libraries for sequencing data quality.

Sample	Clean_Data(bp)	Q20(%)	Q30(%)	GC(%)
M_pool	52302667500	49960429207(95.52%)	46451846247(88.81%)	19371526030(37.03%)
F_pool	51877342500	49912736881(96.21%)	46804330213(90.22%)	19065414992(36.75%)
Wandou35	28405202100	27004018371(95.07%)	25054544392(88.2%)	9928251853(34.96%)
Ruidou1	30204622500	28888901805(95.64%)	26929025839(89.16%)	11297741127(37.4%)

Table S4. The qRT-PCR primers used for verification.

GeneID	F	R
Glyma.02G125100	GGACCATGCGTGCAAAGAAT	ATTTTCCACCACTGGAGGGTC
Glyma.02G125200	GAAGTGGACGGGATGGTGATA	CCCTTTGCACCTTCACTAGGT
Glyma.02G125400	TGCAGTCATCCCAGTTACAGA	AAATTCAAGGCCTTCCCCGC
Glyma.02G125600	TCACCCCATCTCCGAGTTTC	GATCTCTTTCTGGTATATTT
Glyma.02G126000	GGATGAGCGTGAATGGGGAA	AACCAATCACCACCGAGTGC
Glyma.02G126100	ACTGCCACTACCCTCCTTCA	GGTTGAGTTGTTGCTGAGGC
Glyma.02G126300	CAGCGGCACTTGTTTCTGAAC	AGCACTGACTGCAGCAACATC
Glyma.02G126500	GGCTGGACCTGGAGTTGC	CGCGGATCTTCTCCGGG

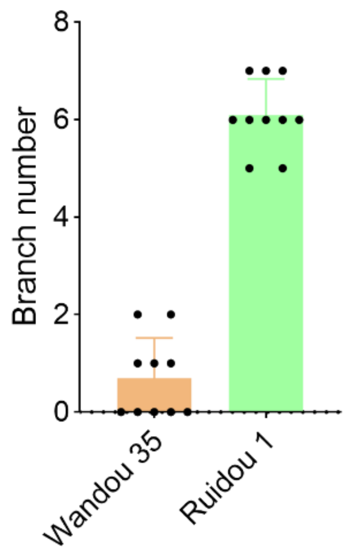

Figure S1 Branch number of Ruidou 1and Wandou 35. Data are shown as mean ± SD, n = 10. Student's *t*-test was used to calculate the *P* values; ***P* < 0.01.

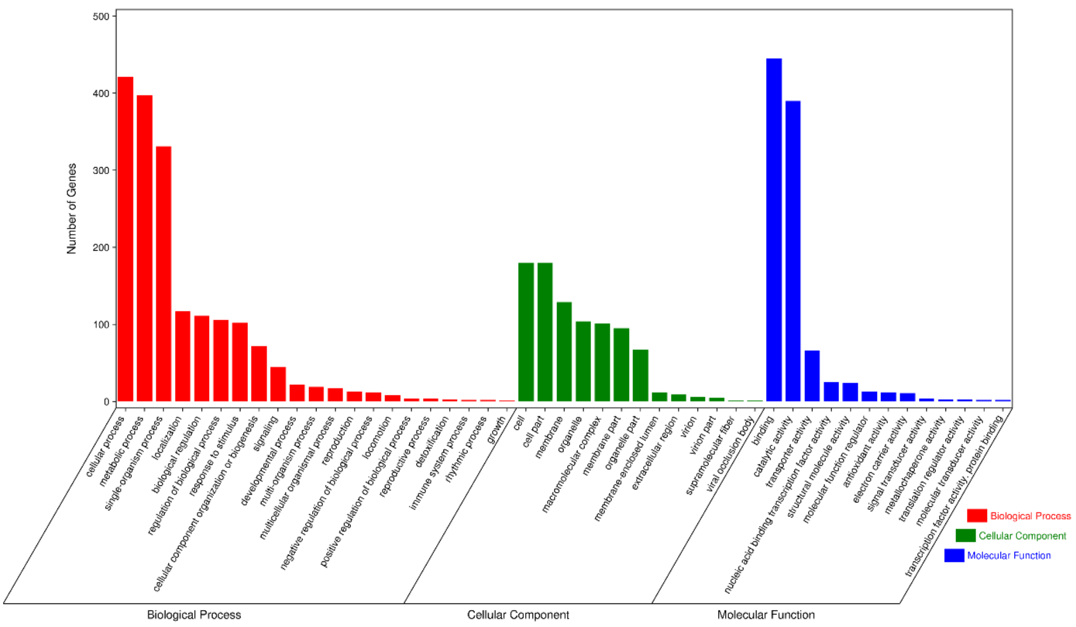

Figure S2 GO enrichment analysis of candidate genes based on BSA results.

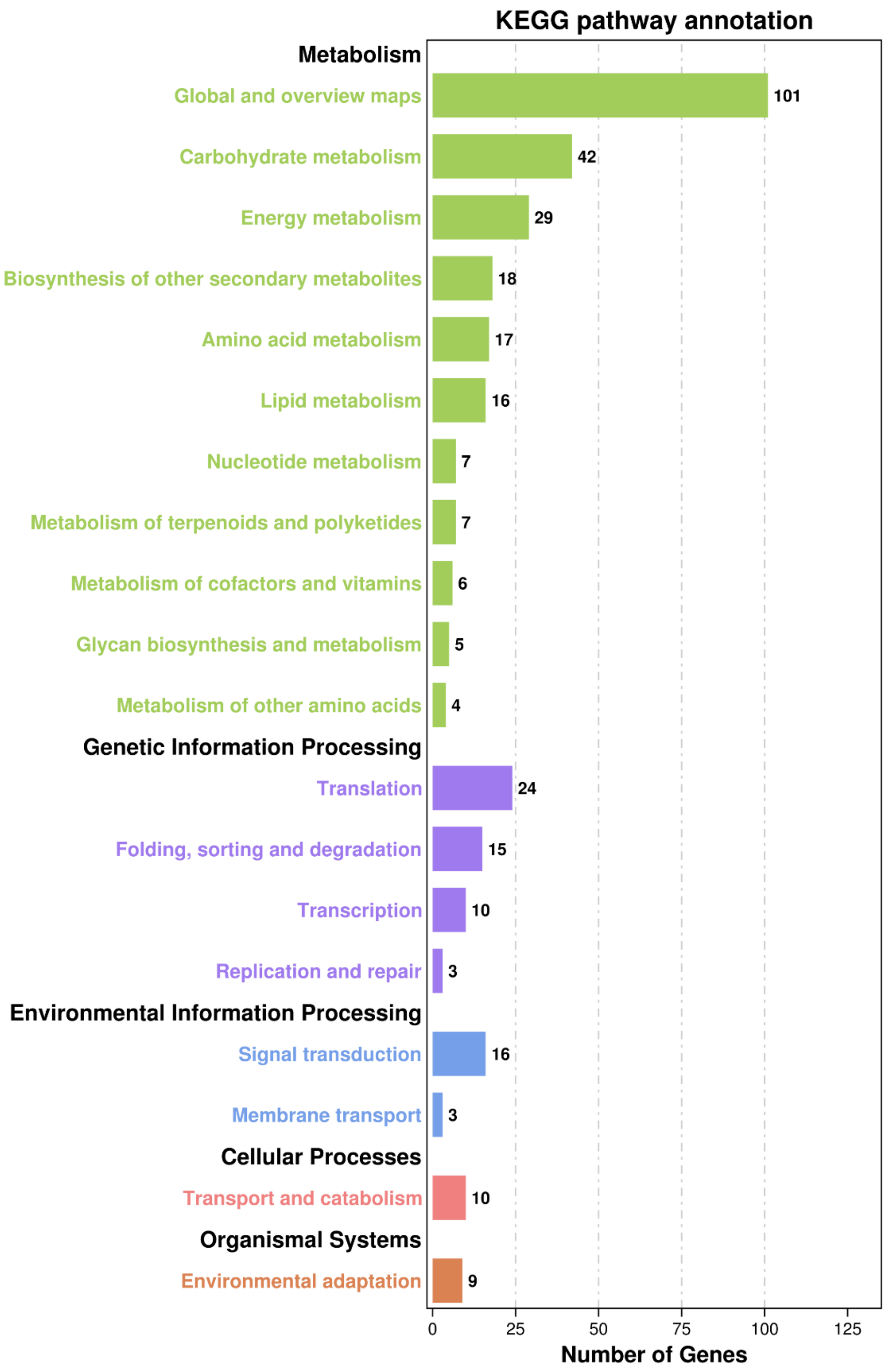

Figure S3 Pathway annotations of candidate genes based on BSA results.
